# Genome-wide survey indicates involvement of loci on canine chromosomes 7 and 31 in patellar luxation in flat-coated retrievers

**DOI:** 10.1186/1471-2156-15-64

**Published:** 2014-05-28

**Authors:** Ineke C M Lavrijsen, Peter A J Leegwater, Chalika Wangdee, Frank G van Steenbeek, Monique Schwencke, Gert J Breur, Freek J Meutstege, Isaac J Nijman, Edwin Cuppen, Henri C M Heuven, Herman A W Hazewinkel

**Affiliations:** 1 Department of Clinical Sciences of Companion Animals, Faculty of Veterinary Medicine, Utrecht University, Utrecht, The Netherlands; 2 Department of Veterinary Surgery, Faculty of Veterinary Science, Chulalongkorn University, Bangkok, Thailand; 3 Dierenkliniek Putten, Roosendaalseweg 162c, Putten, The Netherlands; 4 Department of Veterinary Clinical Sciences, School of Veterinary Medicine, Purdue University, West Lafayette, IN 47907, USA; 5 Steenen Camer 78, Bilthoven, The Netherlands; 6 Hubrecht Institute, The Royal Dutch Academy of Arts and Sciences, University Medical Center Utrecht, Utrecht, The Netherlands; 7 Animal Breeding and Genomics Centre, Wageningen University, Wageningen, The Netherlands

**Keywords:** Patellar luxation, Knee, Dog, Canis, Genome, Association analysis, DNA sequence

## Abstract

**Background:**

Patellar luxation is an orthopedic disorder in which the patella moves out of its normal location within the femoral trochlea of the knee and it can lead to osteoarthritis, lameness, and pain. In dogs it is a heritable trait, with both environmental and genetic factors contributing to the phenotype. The prevalence of patellar luxation in the Dutch Flat-Coated Retriever population is 24%. In this study, we investigated the molecular genetics of the disorder in this population.

**Results:**

Genome-wide association analysis of 15,823 single nucleotide polymorphisms (SNPs) in 45 cases and 40 controls revealed that patellar luxation was significantly associated with a region on chromosome CFA07, and possibly with regions on CFA03, CFA31, and CFA36. The exons of the genes in these regions, 0.5 Mb combined, were analyzed further. These exons from 15 cases and a pooled sample from 15 controls were enriched using custom genomic hybridization arrays and analyzed by massive parallel DNA sequencing. In total 7257 variations were detected. Subsequently, a selection of 144 of these SNPs were genotyped in 95 Flat-Coated Retrievers. Nine SNPs, in eight genes on CFA07 and CFA31, were associated with patellar luxation (P <10^-4^). Genotyping of these SNPs in samples from a variety of breeds revealed that the disease-associated allele of one synonymous SNP in a pseudogene of *FMO6* was unique to Flat-Coated Retrievers.

**Conclusion:**

Genome-wide association analysis followed by targeted DNA sequencing identified loci on chromosomes 7 and 31 as being involved in patellar luxation in the Flat-Coated Retriever breed.

## Background

Patellar luxation (PL) is a common developmental orthopedic disorder in dogs [1-3]. Normally, the patella is located within the trochlea of the femur at a fixed distance from the tibial crest and has only limited sideways movement within this groove [4]. PL occurs when the patella moves out of the trochlear groove and it can lead to degenerative joint disease. Although surgical correction can be performed in most cases, joint cartilage damage may lead to osteoarthritis and consequently to permanent pain and lameness.

Developmental PL is most often seen in small breed dogs [5,6], with the prevalence of PL in breeds appearing to decrease with increasing body size [6,7]. Both medial and lateral PL (inward and outward movability, respectively) occur in dogs, and medial PL is more common than lateral PL in all breeds but one. Lateral PL is less uncommon in large breed dogs than in small breed dogs [5,6,8,9]. The one breed population where lateral PL is more common than medial PL is the Dutch Flat-Coated Retriever (FCR) [10]. In this population, involvement of both hind legs of a dog occurred in half the cases and PL was more common in female than male dogs, as has been reported in other breeds [5,6,11]. The prevalence op PL in Dutch FCR was 24%, much higher than the prevalence reported for presumably mostly American FCR (1.6%) by the Orthopedic Foundation for Animals [http://www.offa.org]. In the Dutch population, lateral PL was diagnosed in 61% of the cases, medial PL in 31% and both lateral and medial in the remaining 8% [10].

The predisposition of certain breeds to PL and the high proportion of dogs with bilateral PL strongly suggests that PL is a heritable trait. The sex predisposition and lack of a Mendelian segregation pattern points to PL being a polygenic disorder [5,6]. The previously calculated heritability of 0.17 in the Dutch FCR population indicated that both environmental factors and genetic factors play a role in the phenotypic appearance of the trait [10]. Chromosomal regions or genes that predispose to PL have not yet been identified.

We report on the genome-wide association analysis of PL in the Dutch FCR population followed by massive parallel DNA sequence analysis and identified loci on CFA07 and CFA31 as having a major influence on the phenotype. This is the first step toward identifying genes that are involved in the development of PL and may help us gain insight into the etiology of this crippling disorder.

## Results

### Genome-wide association analysis

**Figure 1:**
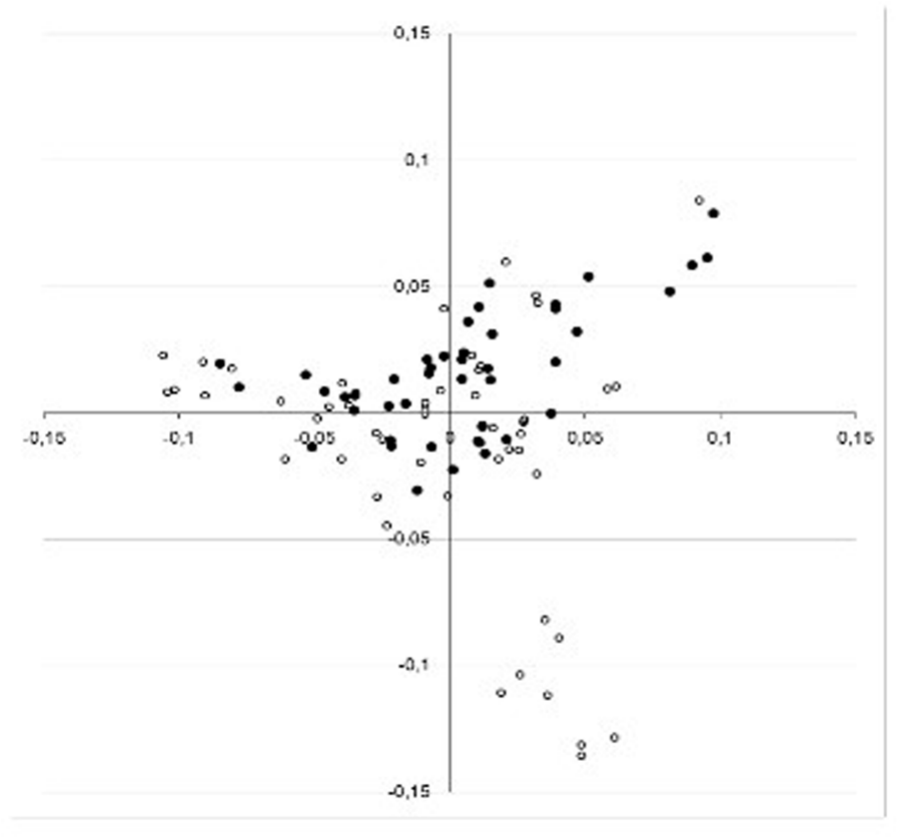
**Identical-by-state plot of Flat-Coated Retrievers.** The 93 dogs were genotyped using arrays for 22,000 SNPs. The first two principal components of a multidimensional identical-by-state matrix of 45 dogs with patellar luxation (filled symbols) and 48 control dogs that were negative for patellar luxation (open symbols) were calculated with PLINK software. The cluster of 8 controls at the bottom right part of the plot was excluded from further analysis.

More than 22,000 SNPs were genotyped in 45 Flat-Coated Retrievers with signs of PL and 48 control dogs of the same breed. In total 15,823 SNPs passed quality control, and these were used to construct an identical-by-state (IBS) plot, based on the first two principal components of the multidimensional IBS matrix (Figure 1). Eight control samples that deviated from the main population were excluded from further analysis.

**Figure 2:**
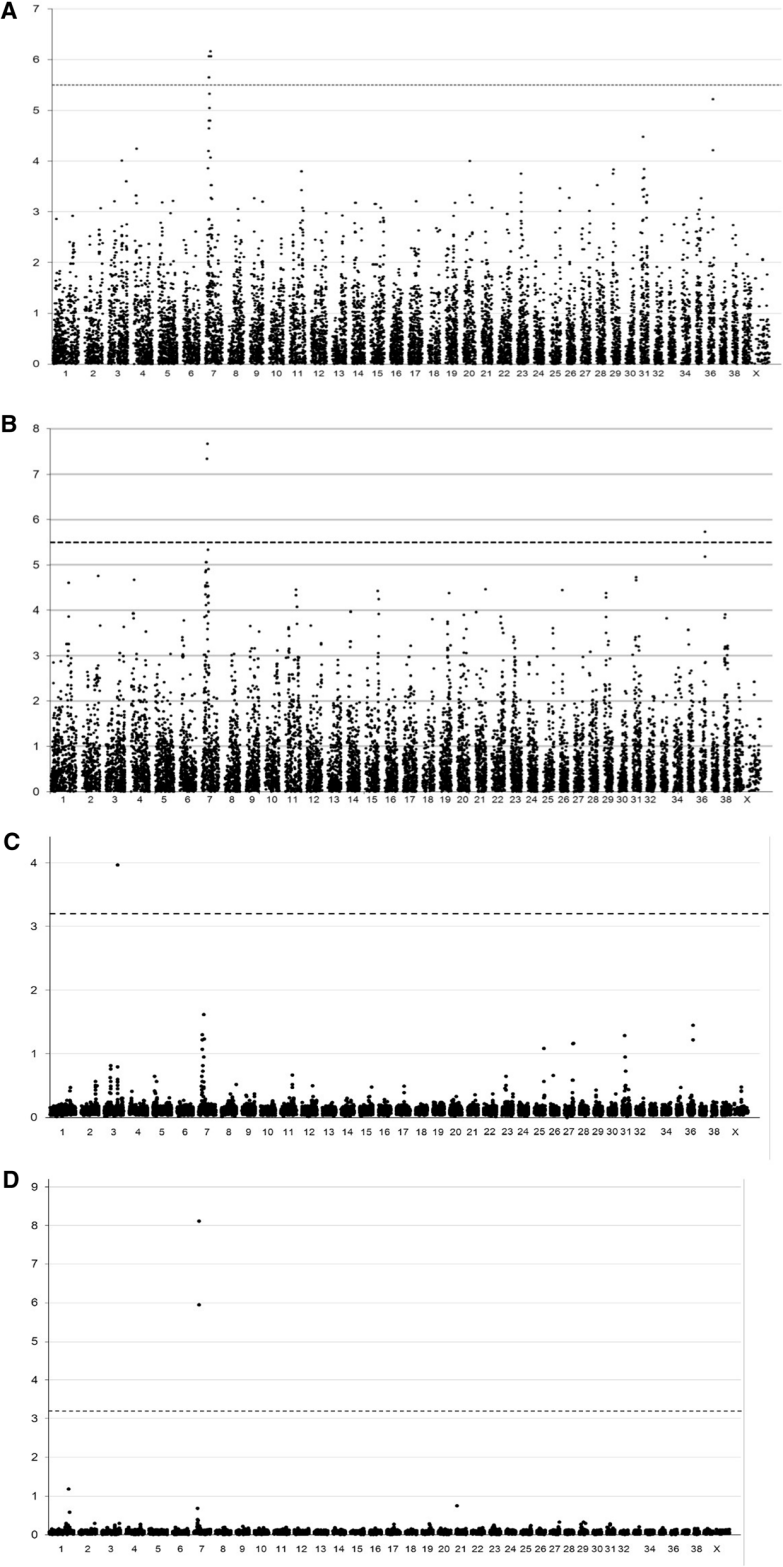
**Genome-wide association analysis of patellar luxation in Flat-Coated Retrievers. (A)** Association of individual SNPs with patellar luxation was analyzed with PLINK software by comparing allele frequencies in the cases (n = 45) and controls (n = 40). **(B)** Association analysis of individual SNPs using the Estimated Breeding Values (EBV) as phenotype using PLINK. **(C)** Multi-SNP association analysis was performed using the case/control patellar luxation status and iBAY software. **(D)** same as **(C)** using EBV as phenotype. The –^10^log of the P-values obtained of individual SNPs are plotted in **(A)** and **(B)**, with the dotted lines indicating the Bonferroni threshold over 15,823 SNPs (α = 0.05). Multi-SNP association values are presented as Bayes factors in **(C)** and **(D)**, with the dotted lines indicating the ‘substantial’ effect threshold according to guidelines by Kass and Raftery [19].

The SNP data were analyzed using two phenotypes and two statistical approaches. A discrete case–control phenotype was alternated with estimated breeding values (EBVs) for individual animals. A χ^2^ analysis of the allele distribution of individual SNPs in PL-positive and PL-negative dogs indicated that PL is associated with a region on chromosome CFA07 (Figure 2A, Table 1). The uncorrected P-value was 6.9*10^-7^ and the empirical P-value after correction for multiple testing using permutations of the genotype data over the groups of dogs was 1.0*10^-3^; the Bonferroni corrected P-value was 1.1*10^-2^.

**Table 1:** Comparison of top SNPs associated with patellar luxation defined as binary trait or by estimated breeding value*

			**MAF**		**Single-SNP**		**Multi-SNP**	
**Chr**	**BP**	**SNP**	**Cases**	**Controls**	**PL status**	**EBV**	**PL status**	**EBV**
1	99888625	BICF2S2314252	0.42	0.23	2.20	2.94	0.42	1.18
3	67056782	BICF2P309055	0.16	0.43	4.01	2.90	3.97	0.22
4	16996349	BICF2S23034244	0.17	0.45	4.24	4.67	0.41	0.16
7	17648777	BICF2S2293048	0.18	0.46	4.20	2.77	0.65	0.04
7	18970233	BICF2G630553500	0.12	0.41	4.79	4.54	0.57	0.21
7	19071723	BICF2P1448362	0.14	0.44	4.64	4.34	0.39	0.26
7	19746349	BICF2G630553889	0.12	0.46	6.06	4.83	1.30	0.69
7	20109002	BICF2P1333659	0.13	0.46	5.65	4.52	1.07	0.31
7	20145907	BICF2P1335550	0.13	0.46	5.65	4.52	1.22	0.40
7	21065761	BICF2S23030368	0.13	0.45	5.33	4.87	0.81	0.31
7	22157845	BICF2P233561	0.19	0.51	5.05	4.11	0.73	0.20
7	23113211	BICF2P1060266	0.12	0.41	4.79	5.06	0.26	0.37
7	24186445	BICF2P424667	0.12	0.41	4.79	5.06	0.33	0.24
7	25490867	BICF2P205579	0.23	0.53	4.07	3.88	0.46	0.15
7	27099172	BICF2S2457585	0.11	0.45	6.16	7.34	1.62	8.11
7	28293222	BICF2P1386712	0.12	0.46	6.06	7.67	1.23	5.95
7	32710038	BICF2G630555333	0.26	0.49	2.73	5.33	0.09	0.14
25	49858895	BICF2P1461096	0.24	0.49	3.01	3.49	1.08	0.19
27	43484050	BICF2G630153501	0.58	0.33	3.02	1.98	1.16	0.17
27	46605159	BICF2G630154851	0.58	0.34	2.77	2.74	1.17	0.33
31	15166531	BICF2S23135348	0.36	0.09	4.48	3.35	1.29	0.12
36	29549762	BICF2S22944651	0.51	0.21	4.22	5.73	1.22	0.07
36	29608881	BICF2G630757990	0.64	0.29	5.22	5.17	1.45	0.09

*MAF: minor allele frequency; Single-SNP associations are presented as –logp values; Multi-SNP associations as Bayesian factors.

Quantitative trait association analysis of individual SNPs with PLINK software and using the EBVs of the 85 dogs instead of their PL status again indicated PL to be associated with CFA07 (uncorrected P-value = 2.2 × 10^-8^) and CFA36 (uncorrected P-value = 1.9 × 10^-6^). Both associations were significant after correction for multiple testing using the Bonferroni correction (Figure 2B).

Multiple SNP analysis using a Bayesian variable selection method [12], as implemented in iBay software, revealed regions associated with PL (Bayesian factor > 1) on chromosomes 3, 7, 25, 27, 31, and 36 when PL was considered as a binary trait (Figure 2C, Table 1). The region on CFA03 explained the largest part of the phenotypic variation (Bayesian factor = 3.97). When the EBV was used as phenotypic score, chromosomes 1 and 7 were associated with the PL phenotype (Bayesian factor >1, Figure 2D, Table 1).

Four regions of interest were selected on the basis of the results from the individual and multiple SNP association studies for both phenotypes. These regions included CFA03 (Canfam2 position 64-69 Mb), CFA07 (15–29.5 Mb), CFA31 (13-21 Mb), and CFA36 (27.5-32 Mb).

### Targeted massive parallel DNA sequencing

The exons of all genes in the four candidate regions were selected for microarray-based enrichment and DNA sequence analysis. The total size of the candidate regions was approximately 32 Mb, and we designed enrichment arrays that covered about 0.5 Mb. The selected regions were sequenced in 15 individual dogs with PL and in a pooled sample from 15 controls.

Enrichment probes could be designed for 93% of the target DNA, so that 476,935 base pairs were represented. Approximately 30% of the generated reads could be mapped to the targeted regions. The average coverage in the target regions was about 80 fold. In all, 7257 variations were observed in fragments that were covered at least 10 times in one or more of the cases and at least 10 times in the control pool. The frequency of the reads of the alternate alleles was used as an indication of the allele frequency in the control pool. In total, 407 variations were detected with a coverage of more than 25 reads in at least 10 cases and in the control pool. The difference in the average allele frequency based on the number of reads per allele between the cases and the control pool was more than 10%. The 40 SNPs with the largest difference in frequency between the cases and the pool of controls are depicted in Table 2.

**Table 2:** Top 40 variations associated with patellar luxation derived from DNA sequence data

**CFA**	**Position**	**Alleles**	**Associated allele**	**Frequency cases**	**Frequency controls**	**Frequency difference**
03	67172456	[A/G]	G	0.54	0.33	0.21
07	15554687	[G/A]	A	0.45	0.23	0.22
07	15995236	[T/C]	C	0.55	0.27	0.28
07	17387000	[A/G]	G	0.53	0.31	0.22
07	19204281	[C/G]	G	0.72	0.50	0.22
07	19301203	[T/C]	C	0.82	0.53	0.29
07	19865384	[A/G]	G	0.65	0.36	0.29
07	20790820	[T/C]	C	0.42	0.16	0.26
07	21406148	[C/T]	C	0.26	0.57	0.31
07	22035860	[A/G]	G	0.72	0.44	0.28
07	22172500	[G/A]	A	0.81	0.57	0.24
07	22173886	[G/A]	A	0.84	0.49	0.35
07	22420986	[C/A]	A	0.51	0.26	0.25
07	23548193	[A/G]	A	0.09	0.33	0.24
07	24673491	[G/T]	T	0.27	0.06	0.21
07	24704299	[C/T]	C	0.12	0.46	0.34
07	25534837	[G/A]	A	0.61	0.37	0.24
07	27238943	[G/A]	G	0.37	0.58	0.21
07	28291838	[C/T]	T	0.84	0.47	0.37
07	28294930	[T/C]	C	0.66	0.31	0.35
07	29308525	[C/T]	T	0.75	0.37	0.38
07	29526712	[G/T]	T	0.38	0.17	0.21
07	30605944	[C/T]	T	0.43	0.21	0.22
07	30605965	[G/T]	T	0.39	0.15	0.24
07	31235880	[T/C]	C	0.67	0.29	0.38
07	31855627	[T/C]	C	0.71	0.46	0.25
07	31856484	[T/C]	C	0.65	0.41	0.24
07	31859005	[T/A]	T	0.16	0.44	0.28
07	32013108	[A/G]	A	0.19	0.47	0.28
07	32149996	[T/C]	C	0.63	0.15	0.48
07	32161825	[G/A]	A	0.71	0.35	0.36
07	32162626	[T/C]	C	0.62	0.27	0.35
23	22621361	[G/A]	A	0.28	0.03	0.25
23	22687566	[G/A]	A	0.40	0.16	0.24
31	14341470	[G/A]	A	0.47	0.26	0.21
31	17088962	[G/T]	T	0.71	0.50	0.21
36	27757717	[C/A]	A	0.54	0.28	0.26
36	27770444	[C/T]	T	0.34	0.13	0.21
36	28095751	[A/T]	A	0.13	0.35	0.22
36	29133623	[T/C]	C	0.54	0.33	0.21

### Genotyping of candidate SNPs in a large cohort

A set of 124 SNPs was selected for further analysis on the basis of the differences in allele frequency between cases and controls. Because the SNPs with the greatest difference in frequency were mainly in regions on CFA07, 20 SNPs were added from regions on CFA03, CFA31, and CFA36. The complete set of 144 selected SNPs is listed in Additional file 1. These SNPs were genotyped in a group of 95 FCRs. This was done to expand the dataset and to ascertain the genotype deduced from the read coverage of each allele. This group of dogs largely overlapped, but was not identical to, the group used in the genome wide SNP analysis. This was because only a limited amount of DNA was available for 6 control dogs. These were replaced by other controls and 10 more controls were added. In total, 127 SNPs were reliably genotyped, 30 of which were monomorphic. Single SNP χ^2^ based analysis of the remaining 97 SNPs identified 8 SNPs on CFA07 and 1 on CFA31 that were associated with PL (P-value < 1.0*10^-4^, Table 3); the SNPs associated with PL located on CFA03 and CFA36 were less significant (P-value > 1.0*10^-4^).

**Table 3:** Intragenic SNPs associated with patellar luxation

**CFA**	**Position**	**Alleles**	-**logp**	**Gene**	**Gene ID**	**Effect**	**Gene description**
07	27010438	G/A	5.44	TNR	490334	Synonymous	Tenascin R
07	28294930	T/C	5.08	SERPINC1	480066	Synonymous	Serpin peptidase inhibitor
07	28329409	T/C	4.21	KLHL20	480067	Intronic	Kelch-like 20
07	30605944	G/A	4.21	FMO2	480076	Synonymous	Flavin containing monooxygenase 2
07	30669327	C/T	4.32	FMO6P	490346	Synonymous	Flavin containing monooxygenase 6
07	31856484	G/C	4.02	SELE	403999	Non_Synonymous	Selectin E
07	32149996	C/T	5.35	BLZF1	490354	Synonymous	Basic leucine zipper nuclear factor 1
07	32162626	T/C	5.09	BLZF1	490354	Intronic	Basic leucine zipper nuclear factor 1
31	14864500	T/C	5.35	NRIP1	478385	Synonymous	Nuclear receptor interacting protein 1

We then investigated whether the 8 SNPs on CFA07 that were associated with PL were also polymorphic in other breeds (24 breeds, with 3–4 dogs per breed). Most alleles associated with PL in the FCR breed were also detected in other breeds, with the exception of the synonymous SNP in the *FMO6* pseudogene at position 30669327, which was not common in the other breeds.

## Discussion

In this study, we analyzed the susceptibility of Flat-Coated Retrievers to PL in two ways: we used the PL status of the animals as a binary trait (PL present or absent) and we used the EBV of all dogs as a quantitative trait. The breeding value takes into account all available phenotypic data from relatives and the animal itself and is a better indicator of genetic susceptibility than an animal’s disease status alone. The observation that the EBV approach resulted in more significant P-values than the binary trait approach illustrates the usefulness of the EBV approach, indicating that a region on CFA07 is involved in the development of PL. The level of significance obtained for this complex disorder using a relatively low number of cases and controls suggests that this region is a major determinant of PL. The choice of DNA sequencing strategy was influenced by two considerations. First, there was the large size (9 Mb) of the region on CFA07 associated with PL. By choosing an exon sequencing strategy, not only could the entire associated region on CFA07 be included, but also additional regions. Second, the number of DNA samples that could be sequenced was limited by the small number of DNA barcode addresses available when the study was performed. Only 30% of the reads mapped to the targeted chromosomal regions instead of the minimally expected 60% (19). We have no explanation for this low yield of the used enrichment procedure with genomic hybridization arrays. In unrelated projects, we obtained higher yields with in solution enrichment protocols.

We pooled control samples because we thought that the allele frequency of DNA sequence variants could be established on the basis of their representation in the reads. However, analysis of individually sequenced DNA of the cases indicated that with an average coverage of 80 reads the allele representation was highly variable and therefore an unreliable indicator of the underlying genotype. The availability of more barcodes since then means that the use of pooled DNA samples can be avoided in future studies.

Approximately 25% of the SNPs genotyped using the KASPar assay were monomorphic, which highlights the importance of confirming Next Generation Sequencing results using independent methods.

The function of the extensor mechanism of the stifle joint depends on the proper alignment of the skeletal and soft tissue elements involved, and different anatomical abnormalities that cause malalignment of these elements have been suggested to be the basis of PL. Ventro-dorsal radiographs of the hip and knees of eight Dutch FCRs with PL did not show signs of bony malalignment [Lavrijsen, unpublished data], and therefore the involvement of muscles or ligaments in PL seems more likely, as suggested by others [13].

We identified nine DNA sequence variants in eight positional candidate genes for PL in affected dogs. One of these, *TNR* coding for tenascin R, is a candidate gene for PL, because mutations in one of its paralogues, *TNXB*, are known to cause Ehlers-Danlos syndrome type III in humans (omim:130020). Ehlers-Danlos syndrome is a connective tissue disorder that is characterized by skin hyperextensibility, articular hypermobility, and tissue fragility. In humans, several disease-causing mutations have been identified in genes involved in the development and maintenance of connective tissue. Ehlers-Danlos syndrome type III is associated with recurrent dislocation of the shoulder joint, the temporomandibular joint, and the patella, without any skeletal deformity. The associated synonymous variant in the tenascin R gene on dog CFA07 could affect the expression of the gene by disturbing the splicing machinery or decreasing the stability of the mRNA. In combination with other genetic risk factors, this variant might predispose Flat-Coated Retrievers to PL. However, studies indicate that that human *TNR* is expressed solely in the brain [14], which is not compatible with its involvement in PL.

It should be noted that although we achieved an average coverage of between 80–90 DNA sequence reads per location, not all target regions were covered sufficiently. It is therefore possible that we missed relevant mutations. In addition, as we only analyzed exons and intron/exon boundaries, we cannot rule out that variants in promoter regions or introns contribute to the phenotype. To confirm the involvement of tenascin R in PL in Flat-coated Retrievers, the *TNR* gene needs to be analyzed in a replication cohort of cases and controls. Investigation of the gene in other breeds predisposed to PL may also lead to confirmation of its role. Additional fine-mapping of the other regions on chromosomes 3, 31, and 36 associated with PL may identify more genetic factors involved in the disorder.

## Conclusions

We identified regions on chromosomes 3, 7, 31, and 36 that are associated with PL in the Dutch Flat-Coated Retrievers. Fine-mapping of the region on CFA07 that showed the strongest association led to the identification of a synonymous variant of *TNR* coding for tenascin R. Mutations in the related protein tenascin XB are the cause of joint dislocations in humans. Follow-up is needed to confirm the involvement of the CFA07 region in PL in the Flat-Coated Retriever and possibly other breeds.

## Methods

### Animals

The animals used in this study were part of a FCR cohort (n = 3835) that had been screened for PL as adults between 1990 and 2007. The dogs were investigated in standing position and in lateral recumbency to control the location of the patella and the possibility to luxate or reposition the patella to grade the movability as introduced by Putnam [15], Grade 0: patella is moving inside the trochlear groove and cannot be manually luxated; grade ‘loose’: patella can be manually positioned on the ridge of the trochlear groove but cannot be positioned out of the groove; grade 1: manually luxatable patella with spontaneous repositioning; grade 2: spontaneous luxation with repositioning upon active extension of the stifle; grade 3: constant spontaneous PL which can be manually reduced; grade 4: constant PL which cannot be manually reduced. All dogs included in this study have been graded by a single board certified veterinary orthopedic specialist (FJM) who made use of the above mentioned grading system and included grade ‘loose’ in the group of grade 0, both referred to as ‘PL-negative’.

Pedigree records were available of 3324 of the phenotyped dogs. These were sired by 398 sires and 678 dams. There were 283 grandfathers and 416 grandmothers of the phenotyped animals. An estimated breeding value (EBV) was calculated for those dogs for which pedigree information was available as described previously [10]. The average EBV for 723 cases was 1.71 (ranging from -2.0 to 6.9) and the average EBV in 2600 controls was -0.45 (ranging from -2.7 to 3.5). We calculated EBVs using all dogs and then chose the dogs to be included based on a high or low EBV and their relationship with other dogs in the sample. Selected dogs with a high EBV did not share parents as did the dogs with a low EBV. In the 93 dogs used for genotyping, the average EBV in the 45 cases was 1.96 (ranging from 0.2 to 4.6) and the average EBV in the 48 controls was -1.37 (ranging from -2.7 to 3.5). The dogs used for genotyping were selected on the basis of their PL status, relatedness to other affected dogs, and their EBV. Of the 45 cases, 40 had PL grade 1 (manually luxable patella with spontaneous repositioning), and 5 had PL grade 2 (spontaneous luxation with repositioning upon active extension).

The Dutch FCR Breeders Club provided the addresses of the dog owners, who were contacted by letter, which was also written on behalf of the Breeders Club, to inform them about the project and with the request that they ask their licensed veterinarian to take a 4 ml blood sample from their dog for DNA isolation. The samples were forwarded by the veterinarians. All dogs were privately owned and owneres provided informed consent. The study complied with the conditions of the Dutch ‘Wet op de Uitoefening van de Diergeneeskunde’ (Law on the Practice of Veterinary Medicine) of March 21, 1990. Approval by an ethics committee for the use of the blood samples was not necessary.

### Genotyping and data analysis

DNA was isolated from the samples collected from the 45 PL-positive and 48 PL-negative dogs, using a standard salt extraction method [16]. The Illumina CanineSNP20 BeadChip with approximately 22,000 single nucleotide polymorphisms (SNPs) was used to genotype the 93 dogs. Only SNPs that had a minor allele frequency of more than 1% and that were genotyped in more than 90% of samples were included in the further analysis. PLINK software [17] was used to calculate an identical-by-state matrix between all 93 samples.

Single SNP association analyses were conducted using both the PL status of the animals as a binary trait and the EBV of the animals as a quantitative trait. A χ^2^ based allelic association analysis with 45 cases and 40 controls was performed, as well as linear regression modeling using the EBVs of the cases and controls. The sex of the animal was included as a covariate in the linear regression. Both analyses were carried out using PLINK v1.07 software [17]. A Bonferroni correction was applied to correct for multiple testing (with 15,823 tests), using α = 0.05 as the threshold for significance (*P*-*value* <1*10^-5.5^). Permutations were also performed (EMP2, n = 1000) as a less stringent method than the Bonferroni correction to correct for multiple testing.

The multiple SNP association analysis was based on a Bayesian variable selection method using iBay software [12,18]. To detect associated regions, we used a model that included a polygenic effect as well as all SNPs simultaneously. A priori a mixture model was used, which assumed that all SNPs belonged to one of two normal distributions: the first distribution contained SNPs with little to no effect on the phenotype (most the SNPs were included in this category), and the second distribution contained the few SNPs that did affect the phenotype. Underlying assumptions about the properties of the two distributions were: (1) the first distribution contained 95% or more of all SNPs and the second contained less than 5%; and (2) the first distribution explained 0.5% of the phenotypic variation observed while the second explained 99.5%. Analogous to the computation and use of the Bayes Factor between two models, we used a ‘parameter-wise Bayes Factor’ (BF) as the odds ratio between posterior and prior probabilities for an individual marker to be in either of the two distributions. According to Kass and Raftery [19], a BF value higher than 3.2 is considered ‘substantial’, a BF value higher than 10 as ‘strong’, and a BF value higher than 100 as ‘decisive’.

### Targeted enrichment of genomic DNA fragments and massive parallel sequencing

DNA samples from 15 cases and 15 controls were purified using phenol/chloroform extraction and ethanol precipitation by standard techniques, and the DNA concentration was measured using Qubit® (Invitrogen). DNA from the 15 cases was individually sequenced (1 μg/sample). Equal amounts of DNA from the control dogs were pooled and 1 μg of pooled DNA was used. The 15 individual samples and the pooled sample were sheared by sonication, underwent end-repair and phosphorylation steps, and then adaptors containing barcode addresses were ligated to the resulting fragments and nick translated as described [20]. Fragments were purified, amplified, and hybridized to a custom designed Agilent® Comparative Genomic Hybridisation Microarray. This array was designed to capture all coding exons including 20 bp flanks in the four candidate regions (CanFam2, ensemble57) as well as in the genes *COL15A1*, *THRB1*, *COL6A3*, *FGF6*, *FGF23*, and *WNT5B*. The captured DNA fragments were sequenced using the SOLiD version 4 system. Array design, library preparation, enrichment hybridization and elution, SOLiD sequencing, and mapping of the DNA sequence data were performed at the Hubrecht Institute (Utrecht, the Netherlands) as described [20,21].

### Genotyping of DNA sequence variations

We genotyped selected variations detected by DNA sequencing in 95 FCRs, using the Komparative Allele Specific PCR (KASPar) assay (KBioscience, Hoddesdon, UK). Two differently labeled allele-specific primers and a common reverse primer were designed for each variable position. Oligo extension PCR in the presence of universal fluorescent reporting dyes, in combination with the fluorescence resonance energy transfer (FRET) technique, makes it possible to determine the distribution of the two alleles in the PCR product. Kluster Caller software (KBioscience, Hoddesdon, UK) was used to determine the genotypes.

The multi-breed sample set contained material from 24 different breeds, 4 samples from English Cocker Spaniels, American Cocker Spaniels, Cavalier King Charles Spaniels, West Highland White Terriers, Cairn Terriers, Border Terrier, Airedale Terriers, Welsh Corgis, Kooikerhondjes (Small Dutch Waterfowl Dog), Basset Hounds, Miniature Schnauzer, Giant Schnauzers, Wetterhouns (Frisian Water Dog), Irish Setters, German Short-haired Pointing Dogs, Dobermanns, Boerboels, Bloodhounds, German Shepherd Dogs, Dutch Shepherd Dogs, Golden Retrievers, Labrador Retrievers, Bernese Mountain Dogs, and 3 samples from Chesapeake Bay Retrievers. The previously described KASPar assay was used to determine genotypes.

### Availability of supporting data

SNP data obtained with microarrays are available in the ArrayExpress database under accession number E-MTAB-2040 [22].

## Competing interests

The authors declare that they have no competing interests.

## Authors’ contributions

ICML participated in the design of the study, performed the statistical analysis and the molecular genetic studies, and drafted the manuscript. PAJL participated in the design and coordination of the study and helped to draft the manuscript. CW participated in the molecular genetic studies and in the statistical analysis. FGvS participated in the DNA sequence analysis. MS participated in the sample collection. GJB participated in the design of the study. FJM participated in the phenotyping and the design of the study. IJN designed the DNA sequence analysis and aligned the sequence. EC participated in the design of the study. HCMH participated in the design of the study, the statistical analysis and helped to draft the manuscript. HAWH conceived of the study, participated in the design and coordination and helped to draft the manuscript. All authors read and approved the final manuscript.

## Supplementary Material

Additional file 1 **Evaluation of DNA sequence variations in Flat-Coated Retrievers.** Excel table (.xlsx) showing allele frequencies of 144 selected SNPs in 95 Flat-Coated Retrievers. Click here for file
